# Peer Support Self-Management Intervention for Individuals With Type 2 Diabetes in Rural Primary Care Settings: Protocol for a Mixed Methods Study

**DOI:** 10.2196/47822

**Published:** 2023-09-04

**Authors:** Xuefeng Zhong, Shaohua Li, Meng Luo, Xinyu Ma, Edwin B Fisher

**Affiliations:** 1 School of Health Management Anhui Medical University Hefei China; 2 Shenzhen People’s Hospital Shenzhen China; 3 Peer for Progress, Department of Health Behavior Gillings School of Global Public Health University of North Carolina–Chapel Hill Chapel Hill, NC United States

**Keywords:** type 2 diabetes, peer support, self-management, rural primary care setting, mixed study, rural, primary care, diabetes, diabetic

## Abstract

**Background:**

The increasing prevalence of diabetes is placing important demands on the Chinese health care system. Providing self-management programs to the fast-growing number of people with diabetes presents an urgent need in rural primary care settings in China. Peer support has demonstrated effectiveness in improving self-management for individuals with diabetes in urban communities in China. A priority then becomes developing and evaluating a peer support program in primary care settings in rural communities of China and determining whether it is feasible and acceptable.

**Objective:**

The aims of this study are (1) to evaluate the feasibility and acceptability of a peer support approach to type 2 diabetes self-management in rural primary care settings; (2) to identify enabler and facilitator factors likely to influence the peer support implementation; (3) to provide primary data and evidence for developing a version of the program suitable for a randomized controlled trial in rural primary care settings.

**Methods:**

Three townships will be sampled from 3 different counties of Anhui province as the study setting. Participants will be recruited based on these counties’ local primary care health record system. The peer supporters will be recruited from among the participants. The peer support program will be led by peer supporters who have completed 12 hours of training. It will be guided by primary care providers. The program will include biweekly meetings over 3 months with varied peer support contacts between meetings to encourage the implementation of diabetes self-management. Mixed methods will be used for evaluation. Qualitative methods will be used to collect information from health care system professionals, individuals with diabetes, and peer supporters. Quantitative methods will be used to collect baseline data and data at the end of the 3-month intervention regarding psychosocial factors and self-management practices.

**Results:**

The results will include (1) quantitative baseline data that will characterize type 2 diabetes self-management practices of individuals with diabetes; (2) qualitative data that will identify enablers of and barriers to self-management practices for individuals with type 2 diabetes in rural communities; (3) both qualitative and quantitative evaluation data, after the 3-month intervention, to demonstrate the feasibility and acceptability of the peer support approach for individuals with type 2 diabetes.

**Conclusions:**

Our findings will inform the design of a tailored intervention program to improve self-management among individuals with type 2 diabetes in rural primary care settings. If we find that the peer support approach is feasible and acceptable, we will develop a larger randomized controlled trial to evaluate effectiveness in multiple rural settings in the province.

**International Registered Report Identifier (IRRID):**

PRR1-10.2196/47822

## Introduction

### Background

#### Challenges of the Diabetes Situation in China

Diabetes is a growing public health challenge. Diabetes mellitus (DM) imposes a huge economic burden on national health care systems worldwide, including in China. The prevalence of diabetes among adults in China has reached 11.6%, comprising about 114 million people and making China the country with the largest number of individuals with diabetes in the world [1]. The prevalence of diabetes in rural areas increased from 8.2% in 2008 to 10.3% in 2010. Although the prevalence is still lower than in urban areas, the growth rate is higher than in urban areas [2]. Furthermore, two-thirds of Chinese adults with diabetes have complications, and only 20.3% exhibit satisfactory glycemic control [3]. To improve noncommunicable disease prevention and control in diseases including diabetes, the National Basic Public Health Service Program (NBPHSP) was launched as part of China’s health care reform initiated in 2009. Subsidized by national and local government budgets, the NBPHSP is mainly implemented by community and township health service centers and health stations in urban areas and village clinics in rural areas [4]. Health management of patients with hypertension and diabetes is an integral and pivotal part of the NBPHSP. However, gaps still exist between guidance on modifiable risk factors and field implementation in primary health care settings. Patients with type 2 diabetes blood glucose and related risk behavior interventions have not been sufficiently studied in primary care settings [5]. Chronic illnesses are largely self-managed, with most of the care becoming the responsibility of patients and their families or others involved in the daily management of their illnesses.

#### Diabetes Self-Management

Early users of the term *self-management* included Thomas Creer et al [6] in a 1976 book on the rehabilitation of chronically ill children. Creer and colleagues said that “the term of self-management indicated that the patient was an active participant in treatment.” Since that time, the term has been widely used, mainly in referring to chronic disease patient education programs. Self-management has been defined as referring to “whether one is engaging in a health promoting activity such as exercise or is living with a chronic disease such as asthma, he or she is responsible for day-to-day management.” [7] Prominent approaches emphasize 3 sets of tasks: medical or behavior management, role management, and emotional management [8]. Additionally, 5 core self-management skills are commonly emphasized: problem-solving, decision-making, resource use, forming a patient–health care provider partnership, and taking action [9].

DM is a progressive and chronic condition lasting the remainder of the patient’s life. It is widely recognized that self-management is the cornerstone of DM care and has the potential to prevent relevant complications and improve quality of life [10]. Accordingly, the success of the treatment for DM depends on the ability of the individual to successfully sustain effective self-management behaviors that include taking prescribed medications, following diet and exercise regimens, self-monitoring blood glucose, and emotionally coping with the rigors of living with diabetes [8]. The goals of diabetes self-management are to optimize metabolic control, prevent acute and chronic complications, and optimize quality of life while keeping costs acceptable. The management of type 2 DM (T2DM) has traditionally been provided by health care professionals who focus on medication and lifestyle changes that can improve glycemic control.

A meta-analysis of self-management programs in diabetes by Norris and colleagues [11] found that diabetes self-management education provided by health care professionals is effective for improving clinical outcomes and quality of life, at least in the short term, but that these benefits sharply decline only a few months after the intervention ends. Therefore, while essential, education is generally not sufficient for most patients to maintain these behaviors over a lifetime of diabetes. To avoid such declines in benefits, evidence indicates that interventions should include elements of social support from families, engagement of multidisciplinary health care professionals, and support from others with diabetes. It is useful for the person with T2DM to recruit allies in supporting self-management who understand the condition, other people with diabetes who can share experiences and tips, or professionals who encourage a collaborative relationship in which they offer advice and the individual chooses how best to use it based on their knowledge of their own body and condition [12].

Studies show that without sustained support, many adults will not succeed in managing their condition well, leading to worse health outcomes including expensive hospitalizations and avoidable complications [13]. Among all these approaches to ongoing follow-up, peer support for diabetes self-management has been considered a promising strategy to achieve sound long-term outcomes for diabetes patients.

#### Peer Support for Diabetes Self-Management

Peer support has been formally defined as “the provision of emotional, appraisal, and informational assistance by a created social network member who possesses experiential knowledge of a specific behavioral stressor and similar characteristics as the target population, to address a health-related issue of a potentially or actually stressed focal person” [13]. Peer support may reduce feelings of isolation and loneliness, provide information about access to health services or the benefits of behaviors that positively improve health and well-being, and encourage more positive health practices [14]. Peer support programs have been widely recognized as promising for facilitating self-management behaviors among individuals with T2DM. Evidence demonstrates that the self-management practices of patients with diabetes are influenced by their knowledge, attitude, self-efficacy, and the presence of a supportive interpersonal, community, and policy environment [15]. The peer support approach emphasizes bringing a group of people with the same condition together to share their self-management challenges and address them as a group rather than using a professional leader who may focus purely on education. Peer group sharing and learning increase knowledge, enhance self-efficacy, and change attitudes for self-management practice [16,17].

#### Diabetes Management in Primary Care Settings in China

Since the 1990s, China has initiated diabetes self-management education programs that have included nurse-based information delivery or clinic counseling targeting inpatients. Literature has shown those programs can improve patients’ knowledge and adherence to medication after intensive short-term interventions [18]. However, the beneficial results could not be sustained in the long term because patients lacked self-efficacy, motivation, and ongoing follow-up support [19,20]. Health policy in China has led to diabetes care and management being delivered mainly in local primary care settings. However, it is difficult to provide personalized health management in rural primary-care settings due to the challenges of professional shortages and limited capacity relative to urban settings [21,22]. Moreover, rural populations often live in worse socioeconomic conditions and have lower health literacy. In rural settings, knowledge, adherence to management practices, and blood sugar control are far from meeting national goals [23,24]. Studies have shown, however, that friends, peers, family members, and community lay health workers may all be important supporters of self-management among individuals with diabetes, especially in areas with restricted health resources [25-27]. Additionally, peer support relies on nonhierarchical, reciprocal relationships, which provide a flexible supplement to formal health system services for people with diabetes. In addition, peer support fosters understanding and trust of health care staff among groups who otherwise may be alienated from or have poor access to health care [28,29]. Peer support implemented through primary care is gaining increased interest in urban China [25,30], while few programs exist in rural areas.

Given the needs in rural areas, and the benefits peer support approaches have achieved in urban settings, this project will explore and assess the feasibility and acceptability of peer support approaches in rural areas. We propose to develop community-based peer support for individuals with type 2 diabetes in rural areas in China. In particular, the study is designed to integrate a peer-support approach into local primary care settings. This study outcome will provide valuable information about the effectiveness, acceptability, and feasibility of a program to improve diabetes self-management in rural, resource-limited communities.

### Study Purposes

Peer support is a culturally adapted, complex intervention; evaluating such an intervention presents challenges to researchers. This study will pursue the following objectives: (1) to evaluate the feasibility and acceptability of a peer support approach to type 2 diabetes self-management in rural primary care settings; (2) to identify enabler and facilitator factors likely to influence the peer support implementation; (3) to provide primary data and evidence for developing a version of the program suitable for a randomized controlled trial in a rural primary care setting.

## Methods

### Overview

**Figure 1:**
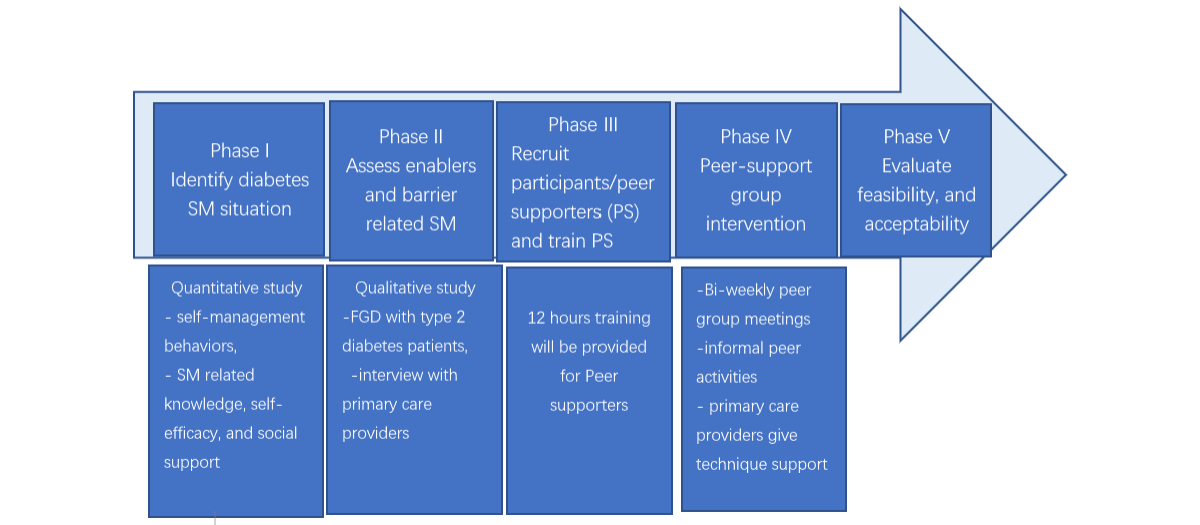
Study phases in detail.

This project will include formative evaluation to develop and then implement and evaluate a peer support approach to improve diabetes self-management in rural primary care. Figure 1 shows each phase in the study.

### Study Setting

This study will be conducted in rural communities in Anhui province. Anhui province is located in eastern China and has about 61 million people. It is divided into 3 geographic areas based on the Huai River and Changjiang River. The 3 areas are north of the Huai River, between the Huai River and Changjiang River, and south of the Changjiang River. These 3 different areas have different customs, cultures, and dietary habits. Stratified sampling will identify 1 county in each of the northern, southern, and middle regions. One township will be randomly recruited in each county. Within each township, the village that has the largest number of adults with T2DM will be selected based on the electronic management system of the local primary care system. This will result in 3 villages representing 3 areas in Anhui province with high numbers of adults with T2DM.

### Participant Recruiting

Study participants will be identified through the Community Chronic Disease Management System (CCDMS), which was established as a national primary care package program in communities in both urban and rural areas. Primary care providers (family doctors and public health physicians) in local primary care settings (township hospitals or village clinics) will contact eligible patients to explain the purpose of the study and details of the program. Since this is an exploratory study with a new intervention approach that has never been tested before in rural Anhui Province, no formal calculation of the sample size will be performed. All patients who are registered in the CCDMS in the sampled study setting (villages) and meet the inclusion criteria will be included if they are willing to participate. An estimated 15 to 20 patients will be recruited in each county, yielding a total sample of 45 to 60 participants. Based on the recommendations of Hertzog [31], a sample of 10 to 40 people is sufficient for a pilot study using a single group to estimate the sample size for a future trial.

### Inclusion Criteria

The inclusion criteria are (1) having T2DM and being registered in the electronic CCDMS in the primary care settings (township hospitals and village clinics), (2) declaring voluntary participation in the study program with the signed informed consent form, and (3) having lived in the study village community at least 6 months.

### Exclusion Criteria

The exclusion criteria include (1) major psychiatric conditions, (2) serious diabetes complications (eg, blindness) that would impede participation in the program, (3) other serious health conditions (eg, terminal cancer), and (4) current enrollment in other research programs.

### Data Collection

Both qualitative and quantitative data will be collected in phases I and II, program development, and phase V, program evaluation. In each phase, there will be 2 stages: first, qualitative data will be collected and analyzed; second, quantitative data will be collected. Here, we describe the general methods of qualitative and quantitative data collection and analysis. The content of these assessments is described in detail in subsequent descriptions of the individual phases.

### Qualitative Data Collection

Researchers will conduct 3 focus group discussions and interviews, with 1 focus group being devoted to each of the following roles: primary care professionals, recruited participants, and peer supporters. Each focus group (6-8 participants) will be led by 2 research team members with a third team member taking notes to be checked for accuracy against audiotapes of the group meetings. Content analyses will identify the common concepts across sites and across roles (patients, professionals, peer leaders). Similar methods will be used to evaluate implementation at the end of the study, also including interviews with patients, peer supporters, and primary care providers.

### Quantitative Data Collection

The questionnaire will be developed by the research team. The questionnaire survey will be administered as face-to-face interviews for self-reported data by trained investigators.

### Quantitative Data Analyses

Social demographic data and health status of the study subjects will be presented as frequencies (percentages) for categorical variables and means (SDs) for continuous variables. Comparison of patients’ characteristics between the 3 settings will be checked by means of 2-tailed *t* tests, *χ*^2^ tests, or nonparametric equivalents. In phase V, the evaluation will include an analysis of differences at 3 months compared to baseline in self-management practice, knowledge, self-efficacy, and perception of social support.

### Ethics Approval and Informed Consent to Participate

The study was approved by the Biomedical Ethics Committee of Anhui Medical University (83220455) on October 13, 2022.

All participants are asked to provide informed consent by responding to the program survey. Participants are informed that consent includes their data being linked to primary care basic program electronic health records and agreeing to surveys and extraction of health record data at the end of the intervention during the evaluation in phase V without additional consent.

Each participant who attends the program will sign a consent form at the start of the intervention. Participants will be informed about the purpose of the study, about the course of the intervention, that it will be conducted in accordance with the study protocol, and that they are free to withdraw from the study at any time. All study data will be deidentified prior to analysis and presented only in anonymized forms.

### Implementation Development

#### Phase I: Understanding the Current Situation of Diabetes Self-Management

Understanding the current situation regarding self-management practice and identifying factors that influence patients’ self-management practice is crucial to developing the intervention program. A study questionnaire will be developed to collect patients’ demographic data, current health status, and diabetes self-management practices. Psychosocial data will be collected by using validated measurement instruments (Table 1). Self-efficacy will be measured by the Chinese Diabetes Self-Efficacy Scale (C-DSES) [32]. Social support will be measured by the Medical Outcome Study Social Support Survey (MOS-SSS) [33], and self-management practices will be measured by the Summary of Diabetes Self-Care Activities (SDSCA) [34].

**Table 1:** Measurement domains and survey tools used at each data collection time point.

Variables	Quantitative measurement instruments	Baseline	3 months
Demographic and health status	Sex, age, ethnicity, education, marital status, occupation (ie, source of livelihood), cohabitants (ie, family members), length of diagnosis with diabetes, complications, and comorbidities	✓	
Psychosocial factors	CDSES^a^, MOS-SSS^b^	✓	✓
Self-management practices	SDSCA^c^	✓	✓

^a^Chinese Diabetes Self-Efficacy Scale.

^b^Medical Outcome Study Social Support Survey.

^c^Summary of Diabetes Self-Care Activities.

#### Phase II: Identify Enablers and Barriers

Health programs and interventions most likely to be successful and achieve desired outcomes are based on a clear understanding of targeted health behaviors, the factors that influence them, and the environmental context in which they occur [13]. It is critical to thoroughly understand the local primary and chronic disease care delivered by family doctors or township public health professionals in rural communities and the factors that might influence the program implementation process. Meanwhile, it is also important to understand the current social network and support for the self-management of individuals with diabetes in the rural context. It is also necessary to know the characteristics of peer group meetings or activities (eg, frequency, duration, place, content, peer leader tasks) that individuals with diabetes, professionals, and peer leaders would find helpful and feasible. The research team will develop interview and focus group discussion guidelines to gather qualitative data regarding these issues. As described above, focus groups will include individuals with diabetes, professionals, and peer supporters. The results will guide development of real-world peer support intervention activities, contents, and delivery methods.

#### Phase III: Peer Supporter Recruitment and Peer Supporter Training

Peer supporters will be recruited by recommendations from both participating patients and village clinic doctors.

##### Inclusion Criteria

The inclusion criteria are as follows: (1) having been diagnosed with type 2 diabetes for ≥1 year; (2) commitment to attending a 12-hour training session at the beginning of the program; (3) commitment to organizing activities with other patients at least 2 times every month; (4) a basic education level, including reading and writing skills; (5) having supportive, nonjudgmental communication skills; (6) willingness to lead group meetings and activities; and (7) willingness to cooperate with primary care providers, including willingness to follow guidelines for when to let primary care providers know about problems participants may be having.

##### Peer Supporter Training

Recruited peer supporters will receive 12 hours of training. Training content will include the following: (1) an introduction to the peer support intervention program in terms of purpose, contents, group activities, and peer leader roles; (2) basic knowledge and skills in diabetes self-management practices; (3) basic health communication skills; (4) the key functions of peer support promoted by Peers for Progress [28], including assisting and encouraging daily diabetes management, providing social and emotional support, linking with community resources and primary care, and providing ongoing support; and (5) guidelines and rehearsal of when and how to seek support from primary care providers regarding participant concerns or problems that are outside their competence.

A peer supporter handbook that includes training materials will be developed for use with training participants.

Because the implementation of peer support needs to be supported within its organizational setting, primary care providers will also receive training related to program implementation, their role in encouraging participation in peer support, and how they can support the peer supporters.

#### Phase IV: Peer Supporter–Led Group Meetings and Activities

Participants will be divided into groups with 6 to 10 people and assigned to peer leaders for 3 months based on how close they live to the peer leaders. Groups will be encouraged to take part in biweekly (ie, every second week) group activities with peer supporters. Peer group members will also be encouraged to organize weekly casual activities such as phone calls, WeChat voice messages, physical exercise groups, and group member family visits.

The biweekly meeting content will be based on findings from phase II and adapted and developed together with primary care providers and peer supporters. The peer group sessions will aim to achieve the following key self-management practice objectives: increasing physical activity; promoting healthy eating habits; ceasing tobacco use; reducing alcohol consumption; adhering to medication; engaging in regular clinical care and diabetes management services provided in primary care settings, such as health education classes, blood sugar and blood pressure monitoring, and physical examinations; and coping with negative emotions.

Peer supporters will also be encouraged to contact participants in their groups between the formal group sessions in order to reinforce the program objectives, update on and discuss the content of missed sessions, and encourage goal attainment and attendance at the next peer group session. These contacts may be informal through meetings within their neighborhoods.

#### Phase V: Evaluation of Feasibility and Acceptability

The aim of this last phase is to evaluate the feasibility and acceptability of the peer support approach in rural primary care settings. Quantitative outcome variables will be measured at baseline and after 3 months to evaluate program feasibility. Qualitative assessments will evaluate the feasibility and acceptability of peer support implementation.

##### Quantitative Evaluation

Key evaluations will be the assessment of differences between baseline and after 3 months (after completion of the peer leader program) in terms of self-management practice, knowledge, self-efficacy, and perception of social support. Additionally, changes will be compared between villages through 2-tailed *t* tests or nonparametric equivalents.

##### Qualitative Evaluation

After 3 months, a descriptive parallel qualitative analysis will be carried out to evaluate the acceptability and feasibility of the program. Semistructured questionnaires and interview guides will be used to collect perceptions of the program from participants, peer supporters, and primary care providers. Questions will include barriers and benefits, individuals’ experiences in the peer-led activities, and suggestions for future program revision and implementation. These will follow the methods for qualitative data collection from peer leaders, professionals, and participants and data analysis described above.

Focus group discussions of participants will be conducted to collect their perceptions, attitudes, experiences, and suggestions for improvement of the peer support program. The focus group discussion and interviews will be audio-recorded and transcribed.

The semistructured interviews with peer supporters will include their experience of the delivery of group interventions, as well as their experience of the process of receiving training and leading group activities. Peer supporters will be encouraged to discuss their feelings and sense of efficacy, the training experience, their motivations, and any positive or negative impacts on their own diabetes management.

## Results

This study was funded in December 2021. Due to COVID-19 restrictions, the project implementation plan for activities in 2022 had to be delayed almost half a year. As of July 2022, we had developed and validated the survey questionnaires and completed interview guidelines. As of January 2023, focus group discussions with both primary care providers and individuals with diabetes had been completed.

## Discussion

### Anticipated Findings

The findings of this study will identify whether a peer support approach is an acceptable and feasible intervention in the rural primary care setting. It will provide evidence on whether the peer support program can enhance uptake, engagement, and maintenance of behaviors to reduce the risk of progression to diabetes. Findings from the study will provide invaluable information on required staff effort and key facilitators and barriers to implementing and sustaining this type of peer supporter–led group program. In addition, it will explore how the peer support self-management approach may be integrated into the NBPHSP in rural areas [4].

Generally, residents who live in the same village are familiar with each other and help each other. Group activities, such as playing mah-jongg and square dancing, are traditional in Chinese culture. This may enhance participation in the groups based on shared experiences and learning from each other. It may also contribute to participants’ knowledge and self-efficacy for self-management through group sharing and activities [35].

There is an accumulating body of studies exploring feasible and acceptable diabetes self-management programs around the world, including those that use peer support. To our knowledge, however, no previous research has examined the feasibility, acceptability, and benefits of peer support for diabetes management in primary care settings in rural China. Thus, the results of this study will provide evidence and insights for developing a peer-led support program for diabetes self-management in this important setting.

We anticipate that the peer support approach for diabetes self-management will be adopted and accepted in rural China, and that participants in a peer supporter–led intervention will show improvement in psychosocial outcomes, adherence to health recommendations in the primary care setting, and better self-management practices. In addition, we hypothesize that the recruitment and training of volunteers with T2DM as peer supporters for participants with the same conditions will be integrated into current chronic disease management practices in primary care in rural areas. We expect that this qualitative research will reveal an in-depth perspective on the impact of this approach, both at the level of the patient as an individual and at the primary care setting level. Successful implementation of this study will provide evidence of the key functions of peer support. Moreover, the results of this study will influence future policies on diabetes management, as well as other chronic diseases in China.

### Potential Limitations and Bias

This study has some limitations. First, outcome measures are primarily self-reported. Second, the study might be subject to selection bias since only voluntary and motivated patients will be included in our study. Third, the study design does not include a control group. The comparison of outcomes before and after the intervention may be biased by social desirability, as well as by other influences.

### Impact and Future Directions

If the peer support approach is feasible and acceptable, we plan to develop the program further and test its effectiveness in a well-powered randomized controlled trial also focusing on the rural setting.

## Abbreviations

C-DSES: Chinese Diabetes Self-Efficacy Scale
CCDMS: Community Chronic Disease Management System
DM: diabetes mellitus
MOS-SSS: Medical Outcome Study Social Support Survey
NBPHSP: National Basic Public Health Service Program
SDSCA: Summary of Diabetes Self-Care Activities
T2DM: type 2 diabetes mellitus

## Data Availability

The data sets generated during and/or analyzed during this study will be made available from the corresponding author upon reasonable request.
